# A traditional way of rice preparation with particular benefits for Arthritis and musculo-skeletal disorders

**DOI:** 10.4103/0975-9476.74421

**Published:** 2010

**Authors:** Muralidhar Kontum

**Affiliations:** *Advisor for S-VYASA, Advisor for Vishwa Mangala Gou Gram Yatra, Bengaluru, India E-mail: rkontham@hotmail.com*

Sir,

The review paper by Chopra in J-AIM[1] concerning Ayurveda drugs for Arthritis is clearly of great value to those who have contracted the disease, but in order to be considered full Ayurveda treatments, such drugs need to be combined with diet and lifestyle recommendations appropriate to the patient. In this sense the studies concerned are not really about Ayurveda as practiced, but about a bowdlerized Ayurveda seen from the biomedical perspective, with its treatments implemented and evaluated strictly as a substitution for western medicine. This constitutes a sad failure to acknowledge Ayurveda’s unique features which promise to give it a highly valued place in medicine globally.

Diet and lifestyle components of Ayurveda are fundamental, and, as is well known, Ayurveda states that even the administration of the correct medicine to cure a complaint may not be of lasting value unless the lifestyle and diet of the patient are attended to. Most chronic illnesses or degenerative diseasees like arthritis result from persistent stresses placed on the system by personal habits which, in the long term, build into unacceptable strain on regulatory function. Over the years, such strain drives the system to breaking point, and pathology results.

Ayurveda is clear that diet and lifestyle have to be attended to. Without such action, say its texts, disease will inevitably return. To implement this, the vaidya plays the role of ‘doctor’ in the true sense of the word’s Latin root, ‘docere’, meaning ‘to teach’. Ayurveda traditional treatments incorporate components of instruction in remedial diet and so on, to help bring the patient’s regulatory system back to whatever state of relative equilibrium can still be regained after years of misuse. If the patient’s system is strong, however, such remedial instruction can often be of actual curative value.

Knowledge of good dietary practices was deeply embedded in India’s traditional culture. Earlier generations often observed age-old procedures for doing particular things for reasons they did not fully understand, but did out of habit from the example of their elders. Skeptical younger generations did not always continue to conform to tradition, and so the knowledge has slowly been lost.

In the case of arthritis, there is a method of cooking rice which appears to be very effective in helping musculoskeletal conditions. The method removes extra starch, increases vitamin content, and generally improves nutritional value, in such a way that arthritic conditions and several other classes of pathology seem to benefit considerably.

The method of preparation is simple and straightforward. First cook your rice with enough excess water, so that when water remaining after cooking is drained off, any excess starch is removed with it. Instead of throwing this valuable starch solution down the drain, it is used constructively: half is offered to animals and plants, while the other half is inoculated with buttermilk and a pinch of fenugreek seeds - apparently because the strain of yeast that grows on fenugreek seeds is of particular value - and fermented overnight. The following day it is added to the pot in which the day’s rice is being cooked.

The value of this procedure is easy to understand. Firstly, the most soluble carbohydrates are removed from the rice, so sugar loading is decreased. Early stages of digestion of ingested material may be expected to cause less impact on blood glucose levels. Secondly, decrease in the food’s Kapha content as a result of reduction of easily available carbohydrates decreases tendencies to constipation, and improves elimination. Finally, the food’s added microbial content from the lactobacillus and yeast considerably increases its nutritional value.

This single procedure therefore has health promoting and preventative value for many conditions. Decrease in sugar loading will reduce tendencies to obesity, insulin resistance, metabolic syndrome, and type 2 Diabetes. The reduction in Kapha and constipation will tend to improve long term colon health, and all conditions involving ama, and amavata, such as the various forms of arthritis. Improved vitamin content from the lactobacillus and yeast (killed by the cooking) will help all conditions. I have extensive anecdotal evidence for all these kinds of benefit from adopting the procedure.

My grandmother never failed to use the above procedure when cooking the day’s rice, and our family enjoyed excellent health, but neither she nor the next generation understood the basis for its benefits. When she died, it ceased. Some years ago, seeking ways to improve health, I remembered her procedure, so I then adopted it, and have found it beneficial.

I recently recommended it to an elderly female friend, who had become bedridden from arthritis. Within two weeks of adopting it she was on her feet and able to do housework again. I then told two vaidyas about it, and after the wife had lost four pounds in ten days, they were sufficiently impressed to print a small pamphlet and distribute it among their patients. They received many reports of freedom from constipation, and feelings of lightness in the body that had developed over a similar time period.

The same vaidyas said that they did not know of reference for this procedure in Ayurveda’s literature, but this does not make it less Ayurvedic. Ayurveda’s knowledge of life and how to improve life-span has been recorded, and ‘codified’, by those who observed benefits received by their fellow human-beings from whatever cause. Today’s Charaka Samhita is a compilation of such observations over many millennia, now amplified by all later Nighantus.

This kind of procedure is a free way to improve health. It could be easily taught by doctor-vaidyas everywhere. Precise assessment of its benefits as a preventive measure would be of interest to evidence-based medicine; so would its therapeutic applications. In his summary of his work to the World Ayurveda Congress*, Dr Chopra mentioned that he would like to see Ayurveda’s holistic treatments assessed, not simply their phytomedicines used as substitutes for biomedical drugs. Might he and his colleagues not consider also evaluating the benefits which the above procedure may bring to his patients, and eventually those in some future arthritis study? Then he will be one step closer to evaluating Ayurveda as actually practiced.

## References

[1] Chopra A, Saluja M, Tillu G (2010). Ayurveda - Modern medicine interface: A critical appraisal of studies of Ayurvedic medicines to treat osteoarthritis and rheumatoid arthritis. J Ayur Integr Med.

